# The World Summit on Sustainable Development: reaffirming the centrality of health

**DOI:** 10.1186/1744-8603-1-8

**Published:** 2005-05-10

**Authors:** Yasmin von Schirnding

**Affiliations:** 1 Sustainable Development and Healthy Environments Cluster World Health Organisation Geneva Switzerland

## Abstract

The World Summit on Sustainable Development (WSSD) was held in Johannesburg in 2002 to review progress since the Rio conference in 1992, and to agree a new global deal on sustainable development. Unlike its predecessor, it was primarily concerned with implementation rather than with new treaties and targets, although a number of new targets were agreed, for example one on sanitation. Failure to agree a target on renewable energy was regarded as a major disappointment of the conference. While relatively modest in its achievements, and with difficulties in achieving consensus in key areas such as energy, trade, finance and globalisation, WSSD nevertheless succeeded in placing sustainable development back on the political agenda, giving new impetus, in particular to the environment and development needs of Africa, with a strong focus on local issues like household energy, water and sanitation. Health was singled out as one of five priority areas, along with water, energy, agriculture and biodiversity, and was devoted a separate chapter in the resulting Plan of Implementation, which highlighted a range of environmental health issues as well as issues relating to health services, communicable and non-communicable diseases. A number of new partnerships were formed at WSSD, including the Healthy Environments for Children Alliance (HECA) launched by WHO, which will form an important platform for implementation. The Commission on Sustainable Development has been designated main responsibility for monitoring and follow up, with its programme of work reorganised to focus on thematic clusters of issues. From the perspective of health, WSSD must be seen as a reaffirmation of the central place of health on the sustainable development agenda, and in the broader context of a process which began in Rio and was given added impetus with the Monterrey Financing for Development conference and the World Trade Organisation meeting held in Doha. Translating policies into action at all levels- global to local – remains the single biggest challenge in the years that lie ahead.

## The world summit on sustainable development: reaffirming the centrality of health

It will be years before we can assess the true impact of the world's largest UN Summit, which brought together governments, the private sector and civil society to agree on a new global deal on sustainable development. Over one hundred Heads of State and Governments addressed the World Summit on Sustainable Development (WSSD) held in August 2002 in Johannesburg, South Africa [1], and over twenty two thousand people participated, including more than ten thousand delegates and eight thousand representatives of NGOs and civil society. It had as its key aims to review progress since the UN Conference on Environment and Development held in Rio in 1992 and to recommend measures to strengthen implementation of Agenda 21 (the global programme of action on sustainable development) and other outcomes of the Rio conference.

The term sustainable development, as originally defined by the 1987 World Commission on Environment and Development [2] (the "Brundtland Commission") was meant to entail "development that meets the needs of the present without compromising the ability of future generations to meet their own needs". It was coined as part of an effort to bring environmental issues into the mainstream of development, recognizing that in order to address the escalating problems related to the environment, the root causes which lay in the broader development process and the global economic system needed to be addressed. As originally articulated "sustainable" captured the environmental issues (assumed to centre on the needs of *future *generations) while "development" captured the economic/poverty issues (assumed to centre on the needs of the *present *generation). The concept has since been broadened, in recognition of the non-environmental aspects of sustainability, and the non-economic aspects of development [3].

In some ways we are only now beginning to judge the success of the Rio conference held ten years earlier. In comparison with the WSSD, which was largely concerned with implementation rather than with new visions, treaties and agreements, Rio led to a dramatic new paradigm shift in thinking on sustainable development, and to new legally binding agreements such as those concerned with biodiversity, and with climate change. Over the ensuing years however, there has occurred a dwindling in high-level political interest and engagement with sustainable development issues.

While WSSD in comparison with Rio was more modest in its immediate achievements, it nevertheless resulted in placing sustainable development back on the political and public agenda. New impetus was given to global action to protect the environment and fight poverty, with the development needs of Africa identified for special attention and support by the international community. A significant departure from Rio was a greater concern with social and economic issues-perhaps not surprising given that the conference was hosted by South Africa. There was also a stronger emphasis on local, as opposed to global, issues – for example with issues such as household energy, water and sanitation, rather than with the global problems associated with climate change which received so much attention in Rio.

The major outcomes of WSSD included a negotiated Plan of Implementation, a Political Declaration and a number of implementation partnerships and initiatives [1]. New targets and agreements were negotiated in a number of important areas, for example in sanitation. Previous agreements such as those relating to the achievement of the Millenium Development Goals [4], were also reaffirmed, making the Johannesburg Plan of Implementation a somewhat eclectic mix of new and past agreements and affirmations, albeit with many important implications for health.

One of the difficulties however was that so many of the key global issues – AIDS, biodiversity, climate change, trade – had their own conference processes, treaty and other mechanisms in place, such that WSSD could only serve to reaffirm these rather than cut new ground. The preparatory meetings preceding Johannesburg were characterized by difficult negotiations and attempts to achieve consensus on key aspects of the plan, particularly on energy, trade, finance and globalization. While consensus was eventually achieved in Johannesburg, contentious issues were often solved by falling back on previously agreed positions.

A separate chapter was included on globalization in the Plan of Implementation (strongly pushed for by the G77/China), which emphasized the need for successful completion of the Doha round of trade negotiations and the implementation of the Monterrey Consensus negotiated at the International Conference on Financing for Development held in Monterrey, Mexico. In addition, the final chapter dealing with means of implementation addressed also health-related commitments related to trade. Issues of contention included the mobilization of financial resources, and the EUs agricultural subsidies. Critics characterized the failure to go beyond Doha to reduce trade-distorting energy and agricultural subsidies in the rich countries as a shortcoming of the summit.

The issue of setting a time-bound target to reverse the trend in natural resource degradation caused disagreement among countries, as did references to the precautionary principle and the ecosystem approach, and the principle of "Common but Differentiated Responsibilities" agreed to in Rio. Other issues which were controversial included debate about the need for stronger governance at the international and national levels (the former emphasized by developing countries and the latter by developed countries), the role of the UN in follow up to WSSD, partnerships and their possible modalities, and the relationship between human rights and environmental protection.

## Centrality of Health

A significant departure from Rio was that health issues featured centrally in WSSD, reflecting increased recognition of health as a resource for, and as an indicator of, sustainable development. Already in 1992 the Rio Declaration stated that "Human beings are at the centre of concerns for sustainable development. They are entitled to a healthy and productive life in harmony with nature." This stressed the important interlinkages between the social, economic and environmental pillars of sustainable development, all of which are underpinned by good health. Further, Chapter 6 of Agenda 21 emphasized the fundamental commitment within sustainable development to "protecting and promoting human health" [5].

At the WSSD there was a greater emphasis on development sectors, and health was singled out by the UN Secretary General as one of five priority issues, in what became known as the "WEHAB" initiative, with emphasis on water, energy, health, agriculture and biodiversity [6,7]. Health was devoted a separate chapter in the negotiated Plan of Implementation [8], and health issues permeated the text throughout. A key message in the wide ranging health agenda at WSSD was that sustainable development cannot be achieved where there is a high prevalence of debilitating illnesses, and the health of the population cannot be maintained without a healthy environment.

Particular emphasis was placed on health issues in relation to environment and poverty concerns. At least a quarter of the global burden of disease can be attributed to environmental factors, many of them poverty-related. The Johannesburg agenda reflected a major shift in recent thinking which has occurred regarding the role of health in poverty reduction and development. Health is far more central to poverty reduction than previously thought, and that realization is now beginning to shape national and global policies [9].

While there were no major breakthroughs in health nor dramatic new agreements reached, a key aspect of significance was recognition of the importance of health in the context of environment, water, energy, agriculture, biodiversity and other issues. Indeed the conference called for a stronger emphasis on health and environment linkages – a move initiated by the Canadians with strong backing from WHO and UNEP.

## Environmental Health Issues

Improving access to safe water, sanitation, clean air, improved waste management and sound management of chemicals were among the key issues which received special attention in Johannesburg. Of particular note was the call for increasing access to sanitation to improve human health and reduce infant and child mortality, prioritising water and sanitation in national sustainable development strategies and poverty reduction strategies where they exist. A new target was eventually agreed, namely to halve by the year 2015 the proportion of people who do not have access to basic sanitation. This new target complements the Millenium Development Goal on access to safe drinking water, and was the subject of much acrimonious debate.

Another important agreement was to aim, by 2020, to use and produce chemicals in ways that lead to the minimisation of significant adverse effects on human health and the environment...taking into account the precautionary approach. Also called for was the promotion of reduction of risks posed by heavy metals that are harmful to human health and the environment.

An agreement to diversify energy supply and substantially increase the global share of renewable energy sources with the objective of increasing its contribution to total energy supply was significant (especially for its health implications), even though the Summit failed to reach agreement on a specific time-bound target. There was also an agreement to improve access to reliable and affordable energy services for sustainable development and resources sufficient to achieve the Millennium Development Goals. Many countries however, viewed the failure to set targets to increase the percentage of the world's power generated by renewable energy sources as the Summit's most significant missed opportunity. The EU reacted by announcing its intention to develop renewable energy sources according to a set timetable with like-minded countries.

Other aspects of note were the call to enhance health education (with the objective of achieving improved health literacy on a global basis by 2010), and an emphasis on capacity building to better assess health and environment linkages. The need to strengthen occupational health programmes was highlighted, as was the necessity of reducing air pollution exposures and related health impacts (including through use of cleaner fuels, modern pollution control techniques and reducing dependance on traditional fuel sources for cooking and heating), as well as controlling lead exposure through eliminating lead in petrol, paints and other sources of human exposure.

## Communicable and Non-Communicable Diseases

Reflecting the knowledge that has accumulated about how ill-health creates and perpetuates poverty, triggering a vicious cycle which hampers economic and social development, the WSSD was also concerned with addressing the main causes of avoidable death in low-income countries. These include HIV/AIDS, malaria, tuberculosis (TB), childhood infectious diseases, maternal and perinatal conditions, nutritional deficiencies and tobacco-related illnesses. There was a strong reaffirmation of targets and goals previously agreed, with emphasis on vulnerable groups such as women and children. While AIDS received due attention, it failed in many respects to be fully recognised as a key development issue at WSSD. This was somewhat surprising given previous concerns that AIDS might dominate the entire WSSD agenda.

A call was made to strengthen the capacity of health care systems to deliver basic health services to all, aimed at improving access to essential drugs, immunisation services, vaccines and medical technology, improving maternal and obstetric care and reproductive and sexual health. These commitments were made with an emphasis on meeting the Millenium Development Goals related to health, including reducing maternal and child mortality. One aspect of the health chapter that proved to be among the most contentious of the Summit and which was of the last to receive agreement on, related to references to "health care and services". There was concern among some that this could be construed to include abortion services. Specific measures to combat and treat HIV/AIDS, malaria, TB and other diseases were called for, with special emphasis placed on the need to mobilise financial resources and to support the Global Fund to Fight AIDS, TB and Malaria.

While many countries continue to see their development efforts hampered by the burden of communicable diseases, at the same time they are faced with the rising incidence of non-communicable diseases (NCDs). The rapid rise of NCDs is also threatening economic and social development as well as the lives and health of millions of people. They represent a major health challenge to global development in the coming century. Low- and middle-income countries suffer the greatest impact, and the rapid increase in these diseases disproportionately affects poor and disadvantaged populations; contributing to widening health gaps between and within countries. In this regard Johannesburg called for programmes to combat non-communicable diseases, mental health, injuries and violence and associated risk factors such as tobacco, alcohol, unhealthy diets and lack of physical activity. In addition, a ten-year framework of programmes in support of regional and national initiatives to accelerate the shift towards sustainable consumption and production (aimed at promoting social and economic development within the carrying capacity of ecosystems) was agreed, with industrialised countries taking the lead.

## The Call for Integrated Strategies and Partnerships

Other aspects emphasised in the Plan of Implementation included the need to integrate health concerns into strategies, policies and programmes for poverty eradication and sustainable development, implementation of the WHO Health for All strategy [10], and creating more effective national and regional policy responses to environmental threats to human health, as well as encouraging health promoting production and consumption policies.

Addressing the underlying determinants of health through intersectoral efforts is key to ensuring sustained health improvements and ecologically sustainable development [11]. In this regard Johannesburg called for increased action from the international community, NGOs, the private sector and local communities to implement sustainable development objectives through partnerships and alliances at all levels- global to local. This represented a major departure from previous UN conferences and was strongly pushed by the US amid initial opposition from many in the G77 bloc, who feared that this would result in an abdication of responsibility away from governments in favour of the private sector and donor interests. Over 220 partnerships (including 16 in health) with 235 million dollars in resources were identified in advance of the summit and around 60 partnerships were announced during the Summit by a variety of countries, with many more announced outside of the formal proceedings.

With health unquestionably recognised as an intersectoral issue the health sector will have to seriously deliberate on its changing role in this complex international landscape. In many parts of the world intersectoral approaches and partnerships have been successfully developed to tackle particular diseases, both communicable (infectious) and non-communicable [3]. Much progress has been made in forging closer links between national health care and other sectors, particularly through local and national intersectoral health and development planning; increased use of planning tools such as health impact assessment procedures; integrated monitoring and surveillance systems; and improved health information systems and indicators [3].

Many countries have instituted new policy and planning frameworks over the past decade, and have developed tools to make health and environment concerns an integral part of the planning process. For example at the national level, National Environmental Health Action Plans have been developed and at the local level, Local Agenda 21 and related activities such as the WHO Healthy Cities Movement, and UN Habitat and UNEPs Sustainable Cities Movement [12] have been important developments.

Effective health, environment and sustainable development policies and programmes depend however on convenient access to information about a large variety of hazards, ranging from biological hazards in food and water, to chemical hazards such as pesticides, to various physical and social factors. This is necessary if health authorities are to effectively discharge their responsibility to protect public health. But it also serves to clarify the extent to which health hazards are attributable to environmental conditions and/or to the activities of sectors other than health.

In general, knowledge of environment and health risks is segmented, and incomplete. Mechanisms to ensure coordination at national, regional and local levels regarding health effects assessment and the development of adequate reporting systems, are commonly lacking. Equally, mechanisms are frequently not in place to ensure that such information, once obtained, is transmitted to the various relevant sectors for action. Integrated databases on development hazards, environmental exposures and health, are urgently required. Well-developed health-and-environment information systems, based on relevant data sets, are essential if scientific monitoring information is to be provided in support of policy and decision-making, planning and evaluation [13]. This is one of the key challenges to the health sector in taking forward the sustainable development agenda.

## The Way Forward

The Summit called on all countries to take immediate steps to formulate national sustainable development strategies and to begin implementation efforts by 2005, with international cooperation supporting the special needs of developing countries. It recommended that Governments immediately enact and enforce clear and effective laws that support sustainable development, develop and strengthen the necessary infrastructure and promote public participation in implementation. Most implementation efforts will take place at the local, national and regional levels, with Governments bearing the primary responsibility.

The main responsibility for monitoring and reviewing progress in carrying out the Johannesburg decisions falls however with the UN Commission on Sustainable Development, which is a functional commission of the UNs Economic and Social Council (ECOSOC). It was set up in 1992 to ensure effective follow-up of the UN Conference on Environment and Development held in Rio. The WSSD called for a strengthened Commission to play a larger role in promoting implementation, including by facilitating partnership initiatives and the sharing of best practices. The Commission has also been charged with developing indicators that will help to determine the state of conditions around the world and will be the basis for discussions on how to overcome obstacles to implementation [14].

The Commission on Sustainable Development has consequently been revitalized and its programme of work reorganised along a multi-year programme of work divided into seven two-year cycles (the first year devoted to a review of progress and the second to policy recommendations), with each cycle focusing on selected thematic clusters of issues. The thematic clusters of issues will be addressed in an integrated manner, taking account of the economic, social and environmental dimensions of sustainable development. Issues being addressed during the first cycle are water, sanitation and human settlements, while in 2006/2007 issues to be addressed are energy, industrial development, air pollution/atmosphere and climate change.

Already there has been much work underway at country level and the various partnerships created have been actively pursuing their respective agendas. This includes the Healthy Environments for Children Alliance (HECA) which was launched by WHO in conjunction with a number of other UN agencies, NGOs and governments [15]. While the Commission has responsibility for tracking partnerships, the partnerships themselves are voluntary and the Commission cannot hold them accountable through the same formal process to monitor government action. The UN has also created new mechanisms to help coordinate efforts in the area of water and energy, namely "UN Water" and "UN Energy" which will help to improve consistency, coherence and cooperation within the UN system.

From the perspective of health, importantly, Johannesburg must be seen as a reaffirmation of the central place of health on the sustainable development agenda [16]. It must also be seen in the broader context of a process which began in Rio and which was given added impetus with the Monterrey Financing for Development Conference and the World Trade Organization meeting held in Doha. The health sector and its allies in other sectors must now muster the necessary resources and commitment to follow through on the sustainable development agenda, and translate policy into action which will ensure a more sustainable path of development for future generations to come.
